# Seismic Target Classification Using a Wavelet Packet Manifold in Unattended Ground Sensors Systems

**DOI:** 10.3390/s130708534

**Published:** 2013-07-04

**Authors:** Jingchang Huang, Qianwei Zhou, Xin Zhang, Enliang Song, Baoqing Li, Xiaobing Yuan

**Affiliations:** 1 Science and Technology on Micro-System Laboratory, Shanghai 200050, China; E-Mails: zhouqianweischolar@gmail.com (Q.Z.); clinging123@sina.com (X.Z.); songenliang@mail.sim.ac.cn (E.S.); libq@mail.sim.ac.cn (B.L.); yuanxb@mail.sim.ac.cn (X.Y.); 2 Shanghai Institute of Microsystems and Information Technology, Chinese Academy of Sciences (CAS), Shanghai 200050, China; 3 University of Chinese Academy of Sciences, Beijing 100049, China

**Keywords:** wavelet packet transform, manifold learning, seismic signal, feature extraction, target classification

## Abstract

One of the most challenging problems in target classification is the extraction of a robust feature, which can effectively represent a specific type of targets. The use of seismic signals in unattended ground sensor (UGS) systems makes this problem more complicated, because the seismic target signal is non-stationary, geology-dependent and with high-dimensional feature space. This paper proposes a new feature extraction algorithm, called wavelet packet manifold (WPM), by addressing the neighborhood preserving embedding (NPE) algorithm of manifold learning on the wavelet packet node energy (WPNE) of seismic signals. By combining non-stationary information and low-dimensional manifold information, WPM provides a more robust representation for seismic target classification. By using a K nearest neighbors classifier on the WPM signature, the algorithm of wavelet packet manifold classification (WPMC) is proposed. Experimental results show that the proposed WPMC can not only reduce feature dimensionality, but also improve the classification accuracy up to 95.03%. Moreover, compared with state-of-the-art methods, WPMC is more suitable for UGS in terms of recognition ratio and computational complexity.

## 1. Introduction

Unattended ground sensor (UGS) systems consist of a lot of sensor nodes and are usually employed for battlefield situation awareness through detection of seismic, acoustic and infrared signals emitted by moving targets [1]. Pedestrians, wheeled vehicles, tracked vehicles and helicopters are the seismic targets of focal interest to land-based monitoring systems, nevertheless they are out of the monitoring capability of radars, thus, UGSs are responsible for detecting and recognizing them. Acoustic sensors and seismic sensors are typically used in UGS systems, however, acoustic target recognition will be affected by weather, Doppler effects and environmental noise. Fortunately, seismic signals are less sensitive to these factors [2], therefore, seismic sensors have become a dominant kind of sensor for UGS systems [3].

However, moving targets' seismic signals are non-stationary [3]. In addition, the seismic signal generated by a moving target is characterized by target velocity, target structure, the signal's propagation distance and local underlying geology, *etc.* [4,5], and is not a simply linear combination of these factors. Therefore, the seismic signal of a moving target is usually considered as a kind of signal with a high-dimensional feature space [4,6,7]. In order to obtain the compact time-frequency feature of each kind of seismic target, it is necessary to reduce correlation contents and fuse the feature set to a minimum but robust one. Thus, a robust seismic feature with the properties of non-stationary and low-dimensional is in urgent demand.

Current feature extraction methods for seismic signals can be classified into three categories, namely, time domain [8,9], frequency domain [4,10,11] and time-frequency domain [3]. On the one hand, time-domain analysis may not be able to recognize targets very accurately because of the interference noise, complicated signal waveforms and variations of the terrain [3]. On the other hand, the accuracy of frequency domain methods may be degraded due to underlying non-stationary in the observed signal [12]. Therefore, recent research has concentrated on time-frequency domain methods (e.g., wavelet transform) thanks to their denoising and localization properties [12].

The conventional seismic target recognition methods usually consist of four steps, just as discussed in [13]. First, the wavelet packet transform (WPT) that overcomes the fixed time-frequency resolution is performed on the original signal. Second, to alleviate the time-variant characteristics of the WPT coefficients, wavelet packet node energy (WPNE) is used as an essential time-frequency feature measure of the target [13]. Although the WPNE provides us with a multi-resolution view of a signal, it still has correlation information. Therefore, the third step is to reduce the dimensionality of the feature space, using some feature selection criteria to discard those feature components which contain little discriminate information, and result in a feature subset having a reduced number of parameters without compromising the classification performance. Finally, the reduced dimensional feature vector is then used as an input to a classifier. In reference [13], even if the feature selection has been already employed to discard redundant information, a complex neural network classifier is still indispensable. In [3], principal component analysis (PCA) is employed to reduce the dimensionality of seismic feature space. Unfortunately, PCA is only suitable for signals whose feature space is globally linear, while seismic features are only locally linear, therefore, the performance obtained in [3] is unsatisfying. Recently, manifold learning has emerged in feature extraction for its capability of effectively identifying low-dimensional structure hidden in high-dimensional data [14–19]. The technique can be realized through several algorithms, including Laplacian Eigenmap [20], locally linear embedding (LLE) [21], and ISOMAP [22], *etc.* These methods yield impressive results on some benchmark artificial data sets, besides some real applications, however, their nonlinear properties make them computationally expensive [23]. Moreover, they can't deal directly with the out-of-sample problem [24,25], which states that only the low dimensional embedding map of training samples can be computed by these traditional manifold learning methods, but the samples out of the training set (*i.e.*, testing samples) cannot be calculated directly, analytically or even cannot be calculated at all [23,26], due to the shortage of a definite mapping matrix. Fortunately, the neighborhood preserving embedding (NPE) algorithm [23] of manifold learning, with a definite mapping matrix, was put forward. Thanks to its specific mapping matrix and excellent merits in dimensionality reduction, NPE may be adequate for seismic pattern recognition.

In this paper, wavelet packet manifold classification (WPMC) is developed for seismic target recognition. Specifically, the WPMC is produced by the three following steps: first, wavelet packet transforms are performed on seismic signals and then WPNE is obtained. Second, a novel feature, wavelet packet manifold (WPM), is obtained by applying the NPE algorithm on WPNE. Third, classification is performed using a K nearest neighbors (KNN) classifier. Since through the combination of manifold learning and wavelet packet transform, distinctive features are obtained, then the classifier is less important and easily implemented. Experiments show the WPMC method not only can reduce the feature dimensionality, but also achieve a satisfying recognition rate. Due to its great advantages in recognition rate and computation consumption, WPMC may be widely used in UGS.

This paper is organized into five sections, including the present one. Section 2 introduces the WPM model. Section 3 illustrates how the WPM feature of seismic targets is more insensitive to environmental variations than other traditional methods and thus suitable for pattern recognition. Section 4 explains how classification is conducted on seismic targets. Finally, conclusions and discussion are provided in Section 5.

## 2. WPM Principle

**Figure 1.**
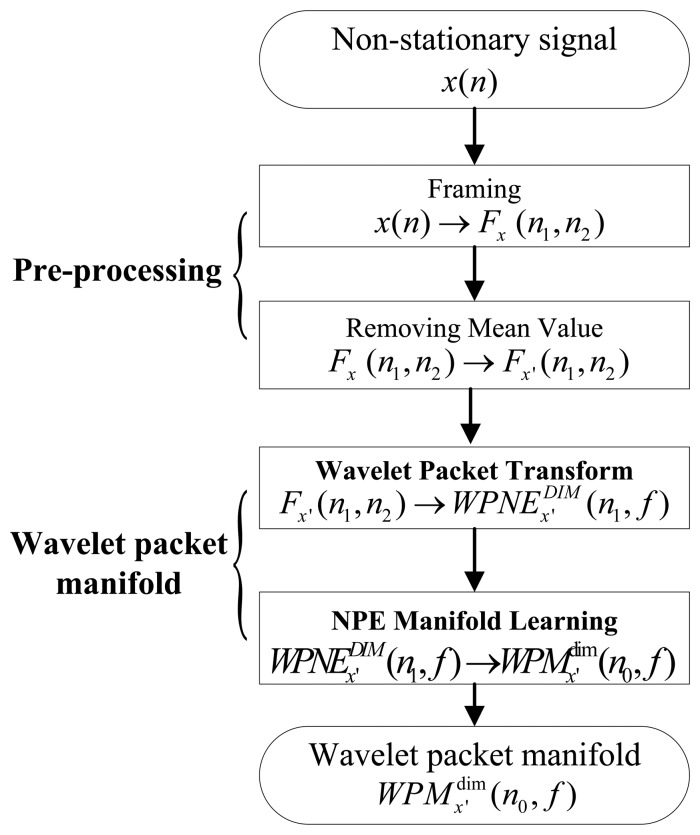
Flowchart of WPM extraction.

It is known that wavelet packet transforms (WPT) are commonly used to reveal the non-stationary characteristics of a seismic target [3,12]. In addition, the NPE algorithm of manifold learning can reveal the low-dimensional structure hidden in high-dimensional data [23]. Therefore, this paper intends to construct a robust feature which is non-stationary and low-dimensional, called WPM, by applying the NPE algorithm to WPNE. To achieve this aim, two techniques comprising WPT and the NPE algorithm of manifold learning are integrated, as indicated in Figure 1. Given an analyzed signal *x*(*n*), the WPM principles are described in the following subsections.

### 2.1. Wavelet Packet Transform

The wavelet transform (WT) possesses good localization performance both in the time and frequency domains [27]. The wavelet packet transform (WPT) is a direct expansion of the conventional discrete wavelet transform (DWT) [28]. In the WPT, both the approximation space and detail space are decomposed to get new lower resolution approximation spaces plus detail spaces. Let *Φ*(*t*) and *ψ*(t) be the scaling function and the corresponding mother wavelet function in the conventional DWT respectively, and $\psi_{0,0}^{0}(\text{t})=\Phi (\text{t})$, $\psi_{0,0}^{1}(\text{t})=\psi (\text{t})$. Using two-scale equations [13], we construct the wavelet packet basis as follows:

(1) $$\psi_{j,k}^{2i}(t)=\sum_{n}h(n)\psi_{j-1,2k-n}^{i}(t)$$

(2) $$\psi_{j,k}^{2i+1}(t)=\sum_{n}g(n)\psi_{j-1,2k-n}^{i}(t)$$

where *i* is the index of node, *j* is the level of decomposition, *h*(*n*) and *g*(*n*) = (−1)^1−*n*^*h*(1 − *n*) are a pair of quadrature mirror filters. The WPT coefficients of a given data *x*(*n*) at the *j*th level and the *k*th point are computed via the following recursive equations:

(3) $$d_{j}^{2i}(k)=\int x(t)\psi_{j,k}^{2i}(t)dt=\sum_{n}h(n)d_{j-1}^{i}(2k-n)$$

(4) $$d_{j}^{2i+1}(k)=\int x(t)\psi_{j,k}^{2i+1}(t)dt=\sum_{n}g(n)d_{j-1}^{i}(2k-n)$$

The decomposition coefficients of the *j*th level can be obtained from the (*j* − 1)th level, finally we can get the coefficients of all levels through sequential analogy. After *j* levels decompositions are accomplished, the frequency ranges of all sub-bands at the *j*th level are:

(5) $$\left\{\left(0,\frac{f_{s}}{2^{j+1}}];(\frac{f_{s}}{2^{j+1}},\frac{2f_{s}}{2^{j+1}}];(\frac{2f_{s}}{2^{j+1}},\frac{3f_{s}}{2^{j+1}}];\cdots ;(\frac{(2^{j}-1)f_{s}}{2^{j+1}},\frac{f_{s}}{2}\right]\right\}$$

where *f*_s_ is the sampling frequency. To alleviate the time-variant characteristics of the wavelet packet coefficients, wavelet packet node energy (WPNE) which measures the signal energy contained in some specific frequency band is adopted. Mathematically, for a discrete signal with frame length as 2*^q^*, the WPNE is defined as Equation (6):

(6) $${\text{WPNE}}_{x'}^{\text{DIM}}(n_{1},i)=\sum_{k=2^{q-j}(i-1)+1}^{2^{q-j}•i}|d_{j}^{i}(k){|}^{2},i=1,2,\cdots ,2^{j}$$

where *n*_1_ represents the index of frames, ${\text{WPNE}}_{x^{'}}^{\text{DIM}}$ subscript *x*′ corresponds to the pre-processing version of original data *x*(*n*) and superscript *DIM* = *N_F_* × 2*^j^* denotes the dimensionality of feature (*N_F_* is the number of frames for every classification operation). The reason why wavelet packet node energy rather than wavelet packet decomposition coefficient is employed as features of target can be found in [13,29].

### 2.2. NPE Manifold Learning

In NPE, searching low-dimensional embedding of high-dimensional space works as follows: given a set of points ***X*** = (**x**_1_, **x**_2_, …, **x***_m_*) in *R^DIM^*, find a mapping matrix ***A*** that transforms these *m* points to a set of points ***Y*** = (**y**_1_, **y**_2_, …, **y***_m_*) in *R^dim^* (*dim* ≪ *DIM*), such that **y***_i_* “represents” **x***_i_*, where **y***_i_* = ***A****^T^***x***_i_*. The computation of matrix ***A*** is divided into three parts.

#### 2.2.1. Constructing an Adjacency Graph

Let *G* denote an adjacency graph with *m* nodes. The *i*th node of graph corresponds to the vector **x***_i_*. In NPE algorithm, K nearest neighbors (KNN) method is adopted to construct *G*.

#### 2.2.2. Computing the Weights

In this step, we need to compute the weights on the edges of *G*. Let ***W*** denotes the weight matrix with *W_ij_* having the weight of the edge from node *i* to node *j*, and 0 if there is no such edge. The weights on the edges can be measured by minimizing the following objective function:

(7) $$\text{W}=\text{arg}\underset{w}{\text{min}}{\sum_{i}\|{\text{x}}_{i}-\sum_{j}W_{ij}{\text{x}}_{j}\|}^{2}$$

with constraints:

(8) $$\sum_{j}W_{ij}=1,j=1,2,\cdots ,m$$

#### 2.2.3. Computing the Projections

In order to compute the projections, we need to solve the following generalized eigenvector problem:

(9) $${\text{XMX}}^{T}\text{a}={\lambda \text{XX}}^{T}\text{a}$$

where:

(10) $$\text{X}=({\text{x}}_{1},\cdots ,{\text{x}}_{m})$$

(11) $$\text{M}={\left(\text{I}-\text{W}\right)}^{T}(\text{I}-\text{W})$$

(12) I=diag(1,⋯,1)

Let the column vectors **a**_0_,…, **a**_*d*−1_ be the solutions of Equation (9), ordered according to their eigenvalues, λ_0_≤…≤λ_*d*−1_ Thus, the embedding is as follows:

(13) $${\text{x}}_{i}\to {\text{y}}_{i}={\text{A}}^{T}{\text{x}}_{i}$$

(14) $$\text{A}=({\text{a}}_{0},{\text{a}}_{1},\cdots ,{\text{a}}_{d-1})$$

where **y***_i_* is a *d*-dimensional vector, and ***A*** is a *N_F_* × *d* matrix. In the NPE algorithm, there are two parameters that need to be configured, namely, the *k* of KNN and output dimensionality *d*. The effectiveness of NPE in dimensionality reduction and target classification is discussed in [23].

### 2.3. Wavelet Packet Manifold

To apply the NPE algorithm on wavelet packet features, first the ${\text{WPNE}}_{x^{'}}^{\text{DIM}}$ of a seismic signal is calculated, then the mapping matrix ***A*** of NPE algorithm is obtained by substituting ${\text{WPNE}}_{x^{'}}^{\text{DIM}}$ for matrix ***X*** (see Section 2.2), finally ${\text{WPNE}}_{x^{'}}^{\text{dim}}$ is achieved by means of multiplying ${\text{WPNE}}_{x^{'}}^{\text{DIM}}$ by ***A***:

(15) $${\text{WPNE}}_{x^{'}}^{\text{dim}}={\text{A}}^{T}\times {\text{WPNE}}_{x^{'}}^{\text{DIM}}$$

where the superscript *T* means the operation of matrix transposition.

Here, the sizes of ${\text{WPNE}}_{x^{'}}^{\text{DIM}}$ and matrix ***A*** are *N_F_* × 2*^j^*, *N_F_* × *d* respectively, therefore the dimensionality of ${\text{WPNE}}_{x^{'}}^{\text{dim}}$ is *dim* = *d* × 2*^j^*. The low dimensional manifold of a new sample can be computed quickly, through multiplying matrix ***A*** by the sample's ${\text{WPNE}}_{x^{'}}^{\text{DIM}}$.

When wavelet packet transform is executed on the seismic signal, each row of matrix ${\text{WPNE}}_{x^{'}}^{\text{DIM}}$ contains the non-stationary information of a specific frequency band. By reducing the row dimensionality of matrix ${\text{WPNE}}_{x^{'}}^{\text{DIM}}$, the non-stationary information and low-dimensional information of a given frequency band is concentrated on the corresponding row of ${\text{WPNE}}_{x^{'}}^{\text{dim}}$. Therefore, the whole matrix of ${\text{WPNE}}_{x^{'}}^{\text{dim}}$ can illustrate the non-stationary and low-dimensional information of target. Consequently, WPM may provide a robust representation for seismic targets.

**Figure 2.**
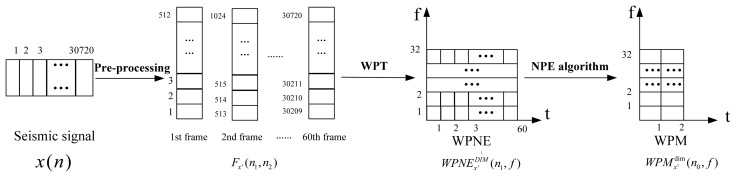
The diagram of dimensionality transformation.

The change of feature dimensionality can be illustrated by an example. If there are 30,720 sampled points and the length of each frame is 512, then the sampled points could be divided into 60 frames. Making five levels wavelet packet transforms on each frame, WPNE, size of 60 × 32, is obtained. Finally, when the NPE algorithm is applied on the WPNE with the parameter *d* chosen as 2, the WPM feature is achieved with the size of 2 × 32, as shown in Figure 2. Through dimensionality reduction, the dimensionality of the target feature is reduced from 60 × 32 to 2 × 32.

## 3. WPM Signature Analysis

WPM with the properties of non-stationary and low-dimensional is analyzed in Section 2, so the main task of Section 3 is to show that WPM is a robust representation for seismic targets. As is known to us, the features of seismic target are strongly influenced by two environmental factors which are environmental noise and environmental underlying geology [5]. Therefore, the aim of this section is to verify that WPM of seismic targets is more insensitive to the variations of environment than other traditional time-frequency features by an experimental approach.

### 3.1. Feature Evaluation

In the evaluation, two parameters including between-class scatter and within-class scatter are employed to quantitatively describe the feature capability in pattern classification. Mathematically, for a given feature set {***f***_1_,……,***f****_Q_*}, where *Q* is the number of feature samples, the two parameters are defined as follows [30]:

(16) $$S_{b}=\sum_{p=1}^{c}(\mu_{f}^{p}-\bar{\mu_{f}}){\left(\mu_{f}^{p}-\bar{\mu_{f}}\right)}^{T}$$

(17) $$S_{w}=\sum_{p=1}^{c}{\sum_{{\text{f}}_{k}\in C_{p}}\left({\text{f}}_{k}-\mu_{\text{f}}^{p})({\text{f}}_{k}-\mu_{f}^{p}\right)}^{T}$$

where $\mu_{f}^{p}$ is the average value of the feature vector for samples with the class *C_p_*(*p* = 1,2,……,c), and $\bar{\mu_{f}}=1/c\sum_{p=1}^{c}\mu_{f}^{p}$ is the total average of the feature vector for all classes. The between-class scatter *S_b_* describes how far different classes are separated, and the within-class scatter *S_w_* indicates how compact each class of samples is distributed. Thus small within-class scatter and large between-class scatter represents better features for classification purpose. Certainly, *S_r_*, the ratio of *S_w_* and *S_b_*, can be taken to characterize the discriminating capability of feature:

(18) $$S_{r}=\frac{S_{w}}{S_{b}}$$

### 3.2. Feature Performance

**Figure 3.**
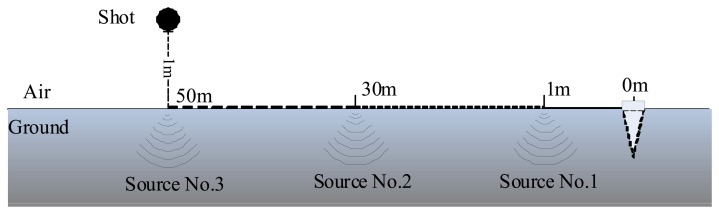
The situation of a shot hitting the ground.

To simplify the exposition, a seismic signal generated by a shot is regarded as the target model. In experiment, the shot falling freely from a height of 1.5 meters hits the ground and induces a seismic signal. The freely falling body motion of the shot is repeated at the same height but three different positions located 1, 10, 30 meters away from the seismic sensor, respectively. The heights of the freely falling body motions are the same, so the strengths of the shots stimulate on the ground are identical. The signal sources produced at three positions are denoted as Source No. 1, Source No. 2 and Source No. 3, separately. HSJ-L1 10-1250, a vertical geophone made by OYO GEOSPACE (Houston, TX, USA), is employed to measure the seismic waves [31], with 1,024 Hz sample frequency. These are no other targets existing in the experimental field when the experiment is executed, so the seismic target is considered as a shot when a bump happens, yet the seismic target is regarded as background noise when no operations are taken. The experimental situation is displayed in Figure 3.

**Figure 4.**
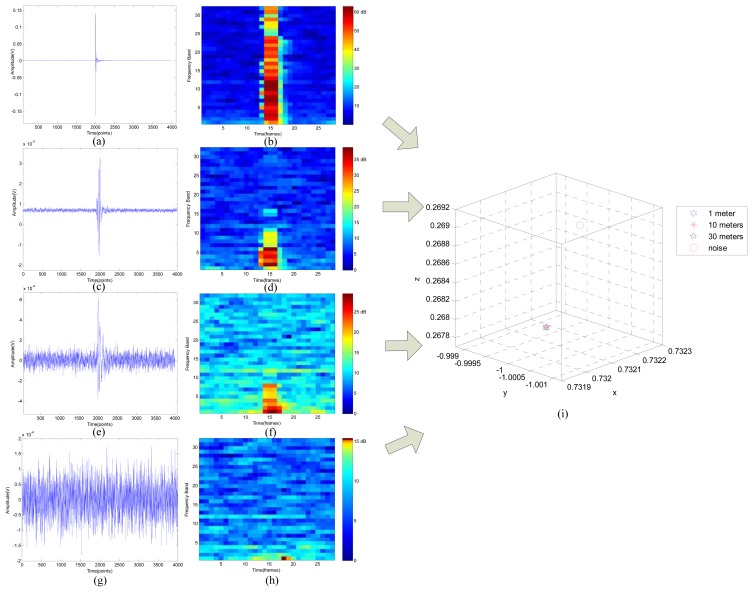
Data set of demonstrative experiments with a shot hitting the ground. (**a**) SS of No.1; (**b**) WPNE of No.1; (**c**) SS of No.2; (**d**) WPNE of No.2; (**e**) SS of No.3; (**f**) WPNE of No.3; (**g**) SS of noise; (**h**) WPNE of noise; (**i**) WPMs of four seismic signals.

The seismic signal (SS), wavelet packet node energy (WPNE) and wavelet packet manifold (WPM) determined in a certain demonstrative experiment which is conducted in a gravel road are shown in Figure 4, where the length of frame is 512 sample points and 128 overlapping points exist in the adjacent frames.

As shown in Figure 4(a,c,e), sharp impulses can only be observed when the shot is hitting the ground, because the sampled seismic signal is induced by background noise when a bump doesn't happen. Examination of Figure 4(a,c,e) shows the gradual degradation of the impulses as the propagation distance increases from 1 meter to 30 meters and the transition of impulses from sharpness to flatness, which means the signal-noise-ratio (SNR) decreases heavily as the propagation distance increases, because the farther signal propagates, the worse the signal degrades. As displayed in Figure 4(a,b), in the moment that impulses occur, the WPNE is obvious different from other times'. According to Figure 4(b,d,f), the WPNEs of impulses become more and more indistinct as the propagation distance increases. Seismic signals are generated in three different positions with different signal-noise-ratios and with different underlying geology conditions, while their signatures, including the waveform in time-domain and WPNEs in time-frequency domain, change considerably when the position of the target varies. In order to combine the non-stationary information and low-dimensional information, the NPE algorithm is applied on WPNE to construct WPM and the dimensionality of WPM is reduced to 3 × 1 (the dimensionality of WPM is first reduced to 32 × 1, subsequently it is reduced to 3 × 1) to clearly display the results as described in Figure 4(i). Fortunately, regardless of the differences of geology condition and environmental noise, the WPMs of the signals generated at the three positions gather at one point, what's more, they are different from noises.

In order to further verify that the robustness of WPM algorithm is better than other traditional time-frequency methods, the above demonstrative experiment was conducted 300 times on three different geologies, specifically the experiment of shot hitting the ground is repeated 100 times in each geology. The *S_r_* of different features which were obtained in the three geologies are detailed in Table 1.

In Table 1, TFM denotes another manifold feature which is calculated by addressing manifold learning on a signal's STFT distribution [14]. According to the above results, the *S_r_* of WPM is the smallest, no matter in which geology condition. Therefore, we can draw a conclusion that WPM, with the properties of non-stationary and low-dimensionality, can capture the pattern difference of seismic targets and be more robust than others, so WPM can be regarded as a kind of feature for target classification.

## 4. Classification Experiment

### 4.1. Experimental Description

**Figure 5.**
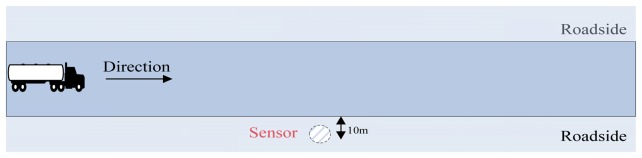
The experimental situation of seismic targets classification.

The underlying geology condition of the experimental field isn't very homogenous, since the field is transformed from a building wasteland and lots of building rubbish remains underground. A seismic sensor is vertically buried 10 centimeters into the ground where 10 meters away from the road. The category of the employed seismic sensor is as discussed in Section 3.2. The experimental situation is shown in Figure 5. In this paper, four categories of seismic targets, including pedestrians, tracked vehicles, wheeled vehicles and helicopters, are involved in our classification, and part of their specifications are listed in Table 2. When the experiment is conducted, the targets move on at a constant velocity, while different targets have different velocities, just as Table 3 shows. Specifically, the helicopter flies at the height of 300 meters above the road and the reason why a helicopter can induce the ground seismic signal can be seen in [2].

### 4.2. Data Sets

Data sets, including training sets and test sets, are sampled in the test field. Each kind of target has 100 samples in the training set and other 50 samples in the testing set. In the experiment, the sampling rate is 1,024 Hz and frame length is 1,024 sample points. There is an overlap of 512 points existing between the adjacent frames. If 5 levels wavelet packet transform is executed on the signal frame, 32 (2^5^ = 32) sub-bands are obtained in the 5th level and the bandwidth of each sub-band is equal to 16 (512/2^5^ = 16) Hz. We make a recognition operation every 60 frames, namely, the size of WPNEs that used in every classification operation is 1,920 (32 × 60 = 1,920).

**Figure 6.**
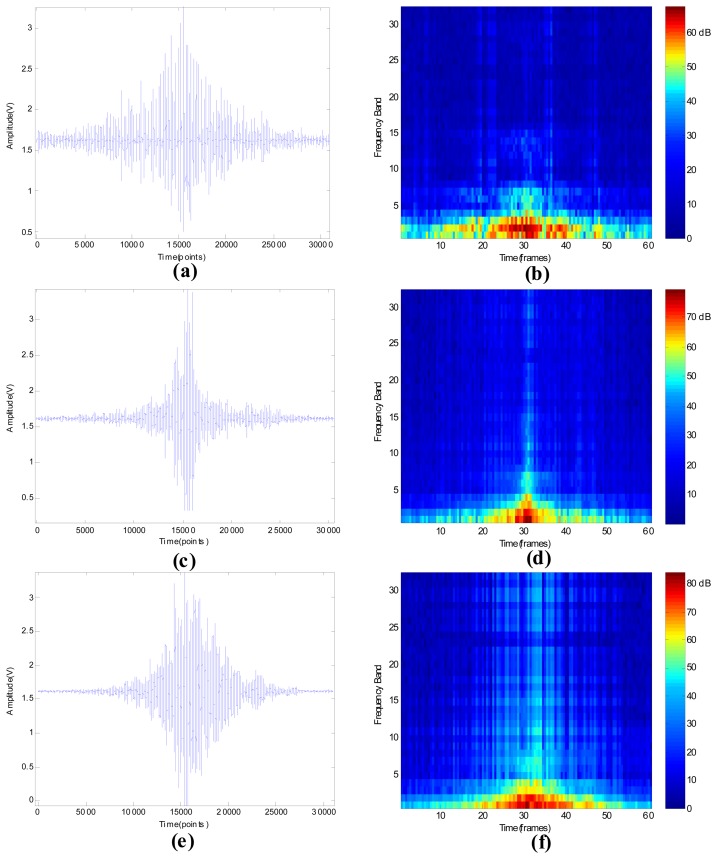

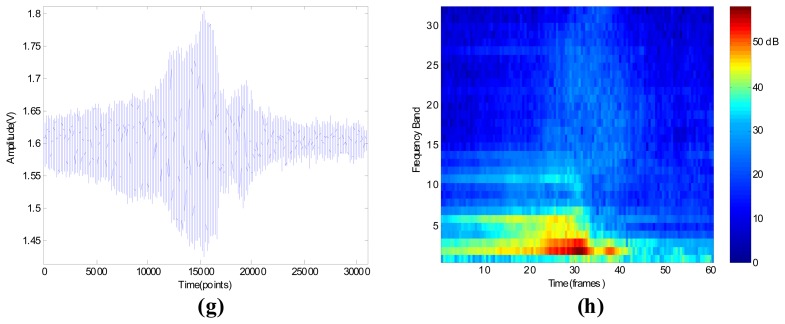
Signal of seismic targets. (**a**) Pedestrian SS; (**b**) Pedestrian WPNE; (**c**) Wheeled vehicle SS; (**d**) Wheeled vehicle WPNE; (**e**) Tracked vehicle SS; (**f**) Tracked vehicle WPNE; (**g**) Helicopter SS; (**h**) Helicopter WPNE.

The obtained seismic signals and WPNEs of seismic targets are depicted in Figure 6. Compared with other ground targets, the envelope of the pedestrian's time-domain signal is the longest and the sparsest because its velocity is the slowest and its impact on ground is the weakest. Among the ground seismic targets, the velocity of the wheeled vehicle is the fastest, so the span of its envelope is the shortest. The velocity of the tracked vehicle is between the former two, but its envelope density is maximal, since its weight is the largest and its stimulation on the ground is the strongest. According to Figure 6(b), only the WPNEs of the first seven frequency bands, corresponding to the signal of 0∼112 Hz, changes significantly when this target is passing through the seismic sensor zone. This phenomenon reveals that the principal frequency component of a pedestrian concentrates around 0∼112 Hz, and it matches well to the conclusion of [32]. The wheeled vehicle, tracked vehicle and helicopter have some differences with the pedestrian, their feature frequencies are higher than pedestrian and as high as 512 (16 × 32 = 512) Hz. Therefore, their WPNEs of all 32 frequency bands will vary obviously when these targets move across the sensor, as shown in Figure 6(b,d,h). The images of Figure 6(b,d,f,h), size of 32 × 60, indicate that the dimensionality of WPNEs is 1,920 (32 × 60 = 1,920). This dimensionality is still too large to classify. Therefore, the manifold of WPNE, WPM, is extracted using the NPE algorithm.

### 4.3. WPM Parameters

The extraction of WPM involves several parameters, such as frame length, decomposition level of wavelet packets, the nearest neighbors' number *k* and output dimensionality *d* of NPE. A nonlinear optimization technique, particle swarm optimization (PSO), is employed to investigate the effect of these parameters on the *S_r_* of WPM. Specifically, the frame length of 256, 512, 1,024, 2,048, 4,096, the decomposition level of 2∼10, the nearest neighbor's number *k* of 1∼50 and output dimensionality *d* of 1∼30 are searched by PSO. The optimal parameters obtained by PSO are listed in Table 4.

### 4.4. Designment of Classifier

The merits of WPM, low-dimensionality and small-scale, determine it is an excellent type of feature for classification. On the basis of WPM, the K-nearest neighbors classifier, which is based on statistical theory and easily implemented, can achieve accurate recognition.

In the KNN classifier, supposing *c* categories exist in the training set, they are *C*_1_, *C*_2_, …, *C_c_*, respectively. Each category has *N_i_* samples, *i* = 1, 2, …, *c*. Euclidean distance $d_{i}^{j}$ that between test sample **X** and each training sample ${\text{X}}_{i}^{j}$ is as follows:

(19) $$d_{i}^{j}=\left\|\text{X}-{\text{X}}_{i}^{j}\right\|,j=1,2,\cdots ,N_{i}$$

where $d_{i}^{j}$ and ${\text{X}}_{i}^{j}$ subscript *i* denotes *c_i_* class, superscript *j* corresponds to the *j*th sample of *C_i_* class. As to an unclassified sample **X**, its *k* nearest neighbors ${\text{X}}_{p}^{j}$ (*j* = 1, 2,…, *k*) are selected out from all training samples according to $d_{i}^{j}$. Finally, the decision function *F_i_* is calculated as follows:

(20) $$F_{i}(\text{X})=\{\begin{array}{ll} F_{i}(\text{X})+0,\;if\;{\text{X}}_{p}^{j}(p=i) & \\ & \left(i=1,2,\cdots c.\;j=1,2,\cdots k.\right)\\ F_{i}(\text{X})+0,\;if\;{\text{X}}_{p}^{j}(p\neq i) & \end{array}$$

If a class satisfies Equation (21):

(21) $$F_{m}(\text{X})=\underset{i=1,2,\cdots ,c}{\max}F_{i}(\text{X})$$

then we can consider that the test sample **X** belongs to the class *m*. That is to say, an unclassified sample **X** is classified by a majority vote of its neighbors, with **X** being assigned to the category that most commonly among its *k* nearest neighbors, after comparing the Euclidean distances between **X** and all training samples.

### 4.5. Classification Performance

**Figure 7.**
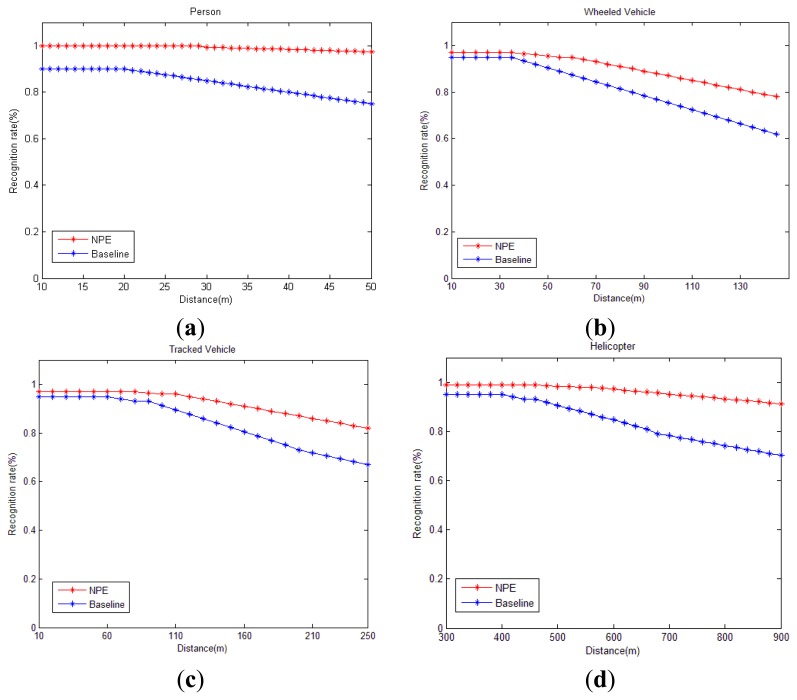
Classification results vary with environment. (**a**) Pedestrian; (**b**) Wheeled vehicle; (**c**) Tracked vehicle; (**d**) Helicopter.

Real-time classification results vary with environment, as shown in Figure 7. The horizontal axis of Figure 7 represents the target position which is denoted by the slant distance between the target and sensor. Naturally, different positions have different signal-noise ratios and different underlying geology conditions. The baseline curve displays the classification results of a method that directly uses WPNE with size of 32 × 60. The NPE curve represents the target recognition rate using WPM whose parameters are as shown in Table 4. In Figure 7, all recognition rates will go down when the distance increases. Fortunately, the NPE curve decreases more slowly than the baseline one. Experimental results denote that the WPM feature is more robust and suitable for moving target classification. Table 5 details the average result of Figure 7. As to the recognition of the four targets, the average classification rate of the NPE approach is 11.53% higher than baseline.

### 4.6. Complexity Analysis

As to a UGS system, the resources of its sensor nodes is very limited. Their complexity of any algorithms developed for the UGS application should be as simple as possible. For the purposes of comparison, we use state-of-the-art methods SDF [12,33] and the algorithm described in reference [32] (called algorithm *A_lgo_*) which are methods for recognizing seismic targets, and implement them independently. The SDF-based feature extraction algorithm mitigates the noise by using wavelet analysis, captures the essential signatures of the original signals in the time-frequency domain, and generates robust low-dimensional feature vectors for pattern classification. Specifically, five symbols are used in SDF and db6 is adapted as wavelet basis by all three algorithms. All of the methods are executed on the Matlab 2011a environment of an industrial computer (dual core, 2.9 GHz-frequency and 2 GB memory) to process the data sets detailed in Section 4.2. The results are shown in Table 6.

According to [12,32] and the above Table 6, on the one hand, the proposed WPMC can process the situation of four targets' classification while the other two algorithms can only do so for only three targets. On the other hand, WPMC achieves higher classification accuracy with lower computation consumption. The results confirm the excellent performance of the proposed WPM feature for seismic targets' classification.

## 5. Conclusion and Discussion

This paper has presented a new WPM signature by combining the wavelet packet node energy and NPE algorithm of manifold learning for a better representation of moving seismic targets. The proposed WPM can not only describe target features in low-dimensional space, but also improve the recognition rate of seismic targets. Furthermore, the advantage of WPM makes it possible that even some simple classifiers, such as KNN, would be good enough for seismic targets classification. The WPM-based classification method enables seismic sensor nodes to carry out precise classification, it does not require the target to be at a certain range from the sensor nodes or a very homogenous underlying geology conditions. Thanks to the excellent merits of WPM in low-dimensionality and feature robustness, the WPMC is very suitable for UGS. Nevertheless, the WPMC is also limited in single target classification due to the complex feature space of mixed targets. In addition, whether the accuracy of classification algorithm will depend on the speed being constant during the classification periods need to be further investigated. These are studies that the authors will focus on in the future.

## Figures and Tables

**Table 1.** *S_r_* of four kinds of feature in three different geologies.

	**STFT**	**WPNE**	**WPM**	**TFM [14]**
Grass	32	21	3	19
Gravel	30.1	22	2	14
Hard Soil	25.2	18.9	1.5	16.4

**Table 2.** Different Targets' Specifications.

**Feature**	**Wheeled Vehicle**				**Tracked Vehicle**	**Low-Altitude Helicopter**

	Car	Truck	SUV	Van		
Weight (kg)	1,425	6,800	1,635	1,713	40,200	3,850
Number of Cylinders	4	6	4	5	10	8
Engine Capacity	78	170	110	140	3,240	1,468

**Table 3.** Targets' velocity.

	**Ground Seismic Source**			**Low-Altitude Acoustic-Seismic Source**
Target Category	Pedestrian	Wheeled Vehicle	Tracked Vehicle	Helicopter

Velocity (km/h)	6	50	40	120

**Table 4.** The optimal WPM parameters.

**Frame Length**	**Decomposition Level**	***k***	***d***
1,024	5	20	1

**Table 5.** Classification results of the four targets.

	**Pedestrian**	**Wheeled**	**Tracked**	**Helicopter**	**Avg.**
Baseline	84.33%	81.45%	84.04%	84.17%	83.50%
NPE	99.13%	92.57%	92.14%	96.35%	95.03%

**Table 6.** Complexity comparison between three algorithms.

	**WPMC**	**SDF**	***A_lgo_***
Algorithm Flow	5 levels wavelet packet transform + NPE algorithm + KNN classifier	wavelet transform + symbolization + SVM	7 levels wavelet packet transform + fuzzy neural classifier
Target Categories	4	3	3
Classification rate	95.03%	90.0%	85.3%
Running time	98 s	120 s	170.2 s
